# Changes in the Transcriptome of Human Astrocytes Accompanying Oxidative Stress-Induced Senescence

**DOI:** 10.3389/fnagi.2016.00208

**Published:** 2016-08-31

**Authors:** Elizabeth P. Crowe, Ferit Tuzer, Brian D. Gregory, Greg Donahue, Sager J. Gosai, Justin Cohen, Yuk Y. Leung, Emre Yetkin, Raffaella Nativio, Li-San Wang, Christian Sell, Nancy M. Bonini, Shelley L. Berger, F. Brad Johnson, Claudio Torres

**Affiliations:** ^1^Department of Pathology and Laboratory Medicine, Drexel University College of Medicine, PhiladelphiaPA, USA; ^2^Department of Biology, Penn Genome Frontiers Institute, University of Pennsylvania, PhiladelphiaPA, USA; ^3^Epigenetics Program, Department of Cell and Developmental Biology, Perelman School of Medicine, University of Pennsylvania, PhiladelphiaPA, USA; ^4^Department of Pathology and Laboratory Medicine, Perelman School of Medicine, University of Pennsylvania, PhiladelphiaPA, USA

**Keywords:** astrocyte senescence, astrocyte function, brain aging, RNA sequencing, brain oxidative stress

## Abstract

Aging is a major risk factor for many neurodegenerative disorders. A key feature of aging biology that may underlie these diseases is cellular senescence. Senescent cells accumulate in tissues with age, undergo widespread changes in gene expression, and typically demonstrate altered, pro-inflammatory profiles. Astrocyte senescence has been implicated in neurodegenerative disease, and to better understand senescence-associated changes in astrocytes, we investigated changes in their transcriptome using RNA sequencing. Senescence was induced in human fetal astrocytes by transient oxidative stress. Brain-expressed genes, including those involved in neuronal development and differentiation, were downregulated in senescent astrocytes. Remarkably, several genes indicative of astrocytic responses to injury were also downregulated, including glial fibrillary acidic protein and genes involved in the processing and presentation of antigens by major histocompatibility complex class II proteins, while pro-inflammatory genes were upregulated. Overall, our findings suggest that senescence-related changes in the function of astrocytes may impact the pathogenesis of age-related brain disorders.

## Introduction

Astrocytes are the most abundant population of cells within the central nervous system (CNS) and the structural diversity and functional complexity of cortical astrocytes is a distinguishing feature of the primate brain (Oberheim et al., 2006). Astrocytes form a functionally coupled network through a series of gap junctions and have pleiotropic roles in maintaining the blood–brain barrier and controlling cerebral blood flow (Abbott et al., 2006); regulating ion, water and neurotransmitter homeostasis (Simard and Nedergaard, 2004); and modulating synaptic transmission as part of the tripartite synapse (Perea et al., 2009). Astrocytes can respond to CNS insults through the acquisition of immune cell features (Jensen et al., 2013), and during repair, astrocytes undergo a spectrum of molecular and functional changes termed reactive astrogliosis (Sofroniew, 2009).

Recently, there has been a paradigm shift toward recognizing the integral role of glial cells in the pathogenesis of age-related cognitive decline and neurodegeneration (Nagelhus et al., 2013; Phatnani and Maniatis, 2015; Pekny et al., 2016). Secreted factors from astrocytes exacerbate the neurotoxicity of amyloid beta (Aβ) in primary culture (Garwood et al., 2011), and contribute to the decline in hippocampal neurogenesis in aged brains (Miranda et al., 2012). Altered astrocyte physiology has also been linked to aging and to the most common age-related neurodegenerative disorder, Alzheimer’s disease (AD), by transcriptome profiling of gene expression changes in astrocytes from aged mouse cortex (Orre et al., 2014) and in glial fibrillary acidic protein (GFAP)-positive cells isolated by laser-capture microdissection from postmortem tissues of subjects with AD (Simpson et al., 2011; Sekar et al., 2015). Therefore, a greater understanding of how aging impacts astrocytes should provide new insight into age-related diseases of the brain.

Aging is the greatest risk factor for cognitive decline and neurodegenerative disease, and a key feature of aging biology that may underlie age-related diseases is cellular senescence. In support of this idea, senescent cells accumulate in tissues with age, including the brain, and at sites of aging-related pathology (Price et al., 2002; Krishnamurthy et al., 2004; Herbig et al., 2006; Bhat et al., 2012; Jurk et al., 2012, 2014; Zhu et al., 2014), undergo widespread changes in gene expression, and demonstrate a pro-inflammatory secretion pattern (Coppé et al., 2008). The induction of senescence in astrocytes has been implicated in neurodegenerative disease (Bhat et al., 2012; Chinta et al., 2013). Either a cell-intrinsic loss of function or the acquisition of detrimental neuroinflammatory function in astrocytes could have profound consequences for the aging CNS. While senescence-associated gene expression changes have been described in cell-types from the periphery that were senescent *in situ* or induced to senesce *in vitro* (Shelton et al., 1999; Gruber et al., 2010), they remain largely understudied in the context of the CNS.

Treatment with sublethal concentrations of hydrogen peroxide (H_2_O_2_) induces senescence in a variety of cell types (Chen et al., 1998; Kim et al., 2011). Our previous studies characterized this type of stress-induced senescence in human astrocytes as determined by changes in cell morphology (enlarged and flattened shape), cessation of division, increased senescence-associated β-galactosidase activity (85% of positive cells compared to 5% of controls), increased expression of p53 and the cyclin-dependent kinase inhibitors p21 and p16^INK4a^, and a p38MAPK-dependent increase in interleukin-6 secretion (Bitto et al., 2010; Bhat et al., 2012). Astrocytes are sensitive to oxidative stress and low doses of H_2_O_2_ are enough to induce the senescence program compared to other cell types (Bitto et al., 2010; Bhat et al., 2012; Aravinthan et al., 2014). This is physiologically relevant because the CNS is particularly exposed to elevated levels of oxidative stress due to several factors including a high metabolic rate with an elevated oxygen consumption compared to its relatively small weight, low antioxidant capacity, and high concentration of lipids and pro-oxidant metals. The generation of this robust oxidative environment disturbs cells and results in oxidative damage to macromolecules, which is a common underlying feature of both aging and diseased brains (Smith et al., 1991; Esiri, 2007; Radak et al., 2011). Levels of mitochondrial H_2_O_2_ and defects in protective mechanisms that reduce it are implicated in cognitive defects in AD mouse models and also in inflammation (Yin et al., 2016).

In order to better understand how astrocyte senescence relates to changes in astrocyte physiology during aging, we investigated global changes in the astrocyte transcriptome using RNA Sequencing (RNA-Seq) following the induction of oxidative stress-induced senescence using H_2_O_2_. From this analysis, we confirmed that senescent astrocytes acquire an inflammatory phenotype indicative of the senescence-associated secretory phenotype (SASP) and downregulate the expression of brain-expressed genes. In keeping with the myriad of complex functions that astrocytes perform in the healthy brain, senescent astrocytes could affect tissue dysfunction during aging and neurodegenerative disease via multiple mechanisms.

## Materials and Methods

### Cell Culture and Senescence Induction

Human fetal astrocytes (passage 1) were obtained from ScienCell Research Laboratories (Carlsbad, CA, USA) and cultured in ambient O_2_ and 5% CO_2_ as previously described (Bitto et al., 2010; Bhat et al., 2012). In order to induce premature senescence via oxidative stress, cells were seeded at standard density (1 × 10^4^ cells/cm^2^) and the following day treated with 200 μM hydrogen peroxide (H_2_O_2_) for 2 h. Cells were considered senescent at least 5 days after the initiation of treatment, as verified previously, (Bitto et al., 2010) and in subsequent quantitative real-time PCR (qRT-PCR) experiments by increases in senescence marker p21, flattened and enlarged morphology, and cessation of division, and were harvested 7 days after treatment. Viability of senescent astrocytes was not significantly different than the controls (92% ± 1 vs. 95% ± 2.7; *p* = 0.08) as measured by the Guava ViaCount assay (EMD Millipore).

### RNA Preparation and Sequencing

Total RNA was isolated using the RNeasy Mini Kit (Qiagen; Valencia, CA, USA) according to the manufacturer’s instructions and the concentration was determined using a NanoDrop ND-1000 spectrophotometer (NanoDrop; Rockland, DE, USA). RNA-Seq libraries were prepared as previously described (Elliott et al., 2013). RNA-Seq libraries were prepared from two replicate cDNA libraries per condition. We used two biological replicates from one donor, where a biological replicate is defined as an independent growth of cells and subsequent analysis, based on the “Standards, Guidelines and Best Practices for RNA-Seq” published by The ENCODE consortium ^1^ recommending the use of a minimum of two biological replicates in RNA-Seq experiments, where a biological replicate is defined as an independent growth of cells and subsequent analysis. The two replicate cDNA libraries per condition (four libraries in total) were submitted to the Next Generation Sequencing Core (NGSC) at the Perelman School of Medicine, University of Pennsylvania, for sequencing. The Illumina HiSeq sequencing platform was used to generate 50 bp single-end sequencing reads. Analysis of RNA-Seq data, including read mapping and differential gene expression analysis using the DESeq package with a Benjamini–Hochberg correction, was performed as previously described by Elliott et al. (2013). The RNA-Seq dataset was deposited in the Gene Expression Omnibus (GEO) at the National Center for Biotechnology under the accession number GSE58910.

### Gene Ontology and Gene Set Intersection Analysis

Gene Ontology (GO) analysis was performed on transcripts that were significantly differentially expressed in senescent astrocytes with a greater than 1.5-fold change and a *p*-value ≤ 0.05 (Benjamini–Hochberg adjusted). The functional annotation clustering tool of the online bioinformatics resource Database for Annotation, Visualization and Integrated Discovery (DAVID) version 6.7 (Huang da et al., 2009) was used to perform GO analysis limited to biological process terms (BP_FAT). By satisfying a false discovery rate (FDR) of 10%, GO terms were considered to be enriched. In addition, all GO categories have a gene count of 10 or greater and a fold-enrichment of 2 or greater. Enriched GO biological process terms were collapsed if they shared 25 or more differentially expressed transcripts and thus considered functionally synonymous. Enrichment (N, B, n, b) is defined as (b/n)/(B/N) where N is the total number of transcripts in the experiment, B is the total number of transcripts within a GO term, *n* is the number of total transcripts in the intersection of the two gene sets in comparison, *b* is the number of transcripts in the intersection that belong to that GO term. “Count” is the number of genes in the single or collapsed GO term. “Weight” is the summed weight of all genes in the GO term, where each gene is given a weight inversely proportional to the total number of GO terms it appears in. “FDR,” i.e., false discovery rate is the percent likelihood of that GO term coming up by the same number of random genes by chance, as calculated by Benjamini–Yekutieli correction of the *p*-value obtained by Fisher’s Exact test. “Genes” represents the genes constituting the GO term. GO analysis on the intersection of senescence and AD downregulated transcripts was done similarly. The enrichment and statistical significance of gene set overlaps between astrocyte and hepatocyte senescence was performed on http://nemates.org/MA/progs/overlap_stats.html, with the number of detected transcripts from the RNA-Seq, 19580, as the total number of genes. In comparison of gene expression in senescent astrocytes to those from AD patient brains, we only compared genes with expression levels in our control set matching those in the Stanford Brain database (Zhang et al., 2014). This criterion was expression level of greater than or less than 100 in both datasets. For this comparison, “Percentage” is “Count” as a percentage of the total number of input genes. *P*-value is a modified Fisher’s exact *p*-value corrected for the representation of the gene set in the whole genome. List total is the total number of genes in the input that are part of any ontology.

### Identification of Transcription Factor Motifs on Differentially Expressed Genes

The chromosomal coordinates of all promoter regions 1000 bp upstream of the transcription start site were obtained using the RefSeq genes track, refGene table and the hg19 human genome assembly on UCSC Genome Browser – Table Browser tool^2^. For all genes up- or downregulated 1.5 fold or more which enriched GO categories, promoter coordinates were submitted to the Cistrome Analysis Pipeline^3^ SeqPos tool. Public motif databases Transfac and JASPAR were searched for motifs enriched in the promoter sequences. Additionally, a *de novo* motif analysis was performed to find motifs with no correlate in the public databases. Results were filtered by human and mouse species-specificity, using a 1000 bp scan length.

### qRT-PCR Validation

Candidate genes were chosen based upon pathways of interest for validation by qRT-PCR. Total RNA was independently isolated as described. Primers were designed using the PrimerQuest design tool to span an exon–exon junction and were supplied by Integrated DNA Technologies (IDT, Coralville, IA, USA). The NCBI Basic Local Alignment Search Tool (BLAST) was used to confirm the specificity of primer sequences. The primers used in qRT-PCR assays are listed in Supplementary Table S1. SYBR Green-based RT-PCR was performed with Verso 1-Step RT-qPCR reagents (Thermo Fisher Scientific; Pittsburgh, PA, USA) on an Applied Biosystems 7500 Real-Time PCR System (Life Technologies, Grand Island, NY, USA). Dissociation curve analysis was performed to verify single products for each reaction. The absence of product in reactions without reverse transcriptase (no RT) was also verified. Data analysis was performed using DataAssist software v3.01 (Life Technologies, Grand Island, NY, USA). The data were glyceraldehyde-3-phosphate dehydrogenase (GAPDH)-normalized and expressed as fold change (RQ) relative to pre-senescent astrocytes.

### Cell Cycle Analysis

Pre-senescent astrocytes (60–70% confluent) and astrocytes treated with H_2_O_2_ to undergo stress-induced premature senescence were grown in complete Astrocyte Medium (AM, ScienCell) as described (Bitto et al., 2010; Bhat et al., 2012), harvested by trypsinization, washed in phosphate buffered saline (PBS), and fixed with ice cold 70% ethanol overnight at 4°C. Fixed cells were centrifuged to remove ethanol, washed with PBS, and stained with Guava Cell Cycle reagent (EMD Millipore; Billerica, MA, USA) containing the nuclear DNA stain propidium iodide (PI) for 30 min at room temperature in the dark. Guava Cell Cycle data were acquired using Guava EasyCyte Mini flow cytometer using the Guava Cell Cycle program (Guava Technologies, Hayward, CA, USA). The percentage of cells in cell debris, G1-, S-, and G2/M-phase of the cell cycle was determined using the ModFit LT curve fitting algorithm, version 4.0.5 (Verity Software House, Topsham, ME, USA).

### Bromodeoxyuridine (BrdU) Incorporation Assay

Pre-senescent astrocytes in log phase of growth and astrocytes that were treated with H_2_O_2_ to undergo stress-induced premature senescence 7 days prior were treated with 10 μM BrdU (5-bromo-2′-deoxyuridine; BD Pharmingen; San Diego, CA, USA) in complete astrocyte medium for 30 min. After this incubation, cells were harvested by trypsinization, washed in PBS, and fixed with ice cold 70% ethanol. Fixed cells were centrifuged to remove ethanol, resuspended in 2N HCl and incubated for 30 min at room temperature for DNA denaturation, neutralized with 0.1 M Na_2_B_4_O_7_ (pH 8.5), and washed two times in PBS containing 5% fetal bovine serum (FBS). Anti-BrdU monoclonal antibody (eBioscience; San Diego, CA, USA) diluted 1:100 in PBS containing 0.5% Tween-20 was applied for 30 min at room temperature, after which cells were washed, and resuspended in goat anti-mouse-Alexa Fluor 488 (Molecular Probes, Life Technologies; Grand Island, NY, USA) diluted 1:100 in 1x PBS containing 0.5% Tween-20 for 20 min at room temperature in the dark and then washed twice with PBS-5% FBS. Cells were stained with Guava Cell Cycle solution as described previously and analyzed using the Guava EasyCyte Mini flow cytometer using the Guava ExpressPlus program and the percent of cells labeled with BrdU was quantified. The percent of BrdU positive cells was quantified using FlowJo software v10 (Tree Star; Ashland, OR, USA).

### Immunofluorescence

Cells were seeded on coverslips and fixed with 4% paraformaldehyde in PBS, permeabilized with PBS containing 0.1% Triton-X-100, and blocked in PBS containing 0.1% bovine serum albumin (BSA) and 5% normal donkey serum for 2 h at room temperature. Coverslips were incubated with rabbit anti-phosphorylated histone H3 (Ser10) (Upstate Biotechnology; Lake Placid, NY, USA) diluted 1:500 in PBS containing 0.1% BSA overnight at room temperature. Following washes with PBS, coverslips were incubated with Alexa-Fluor Donkey 555 anti-Rabbit (Life Technologies; Carlsbad, CA, USA) diluted 1:500 in PBS 0.1% BSA for 1 h at room temperature protected from light. Coverslips were then washed, stained with DAPI, and mounted on slides with Vectashield fluorescence mounting medium (Vector Laboratories; Burlingame, CA, USA). Cells were visualized using an Olympus BX61 fluorescence microscope coupled with a Hamamatsu ORCA-ER camera and using SlideBook software (Intelligent Innovations, Inc., Denver, CO, USA). The percent of cells positive for phosphorylated histone H3 was quantified.

## Results

### RNA-Seq Broad Picture of Differentially-Expressed (DE) Genes

**FIGURE 1:**
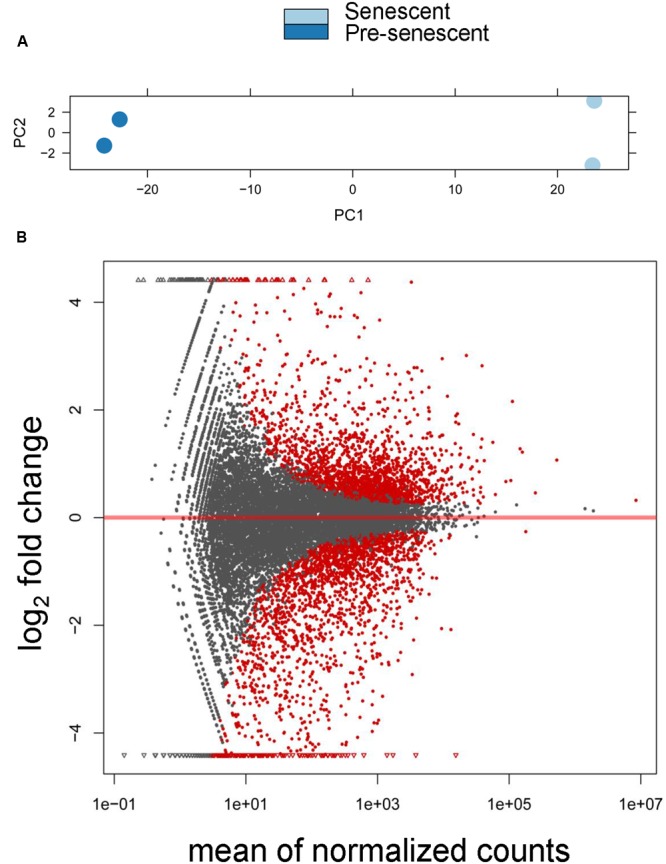
**Differential expression analysis of pre-senescent vs. senescent astrocyte transcriptomes. (A)** Principle component analysis (PCA) revealed the greatest variability in the RNA-Seq data was due to the treatment condition (PC = principle component). **(B)** DESeq Plot showing the expression fold changes compared to pre-senescent and the expression levels of 19520 transcripts (gray) out of which 3569 are differentially expressed transcripts with each symbol representing a single transcript (red symbols are statistically significant at *p* < 0.05 Benjamini–Hochberg adjusted). A positive log_2_ fold change indicates transcripts that are upregulated in senescent cells, whereas a negative log_2_ fold change represents transcripts significantly downregulated in pre-senescent astrocytes. “Mean normalized counts” are the raw read counts, normalized by the total library size, and averaged for each group.

We sequenced two biological replicates each of pre-senescent astrocytes and astrocytes induced to senesce by oxidative stress, and obtained approximately 12 to 35 million reads per sample. From these datasets, ∼97.5% of all reads mapped to the reference human genome (hg 19; see Supplementary Table S2 for all mapped transcripts). We also found by a principle component analysis (PCA) that these samples were tightly clustered based on cellular treatment (**Figure 1A**). Expression levels of genes between the pre-senescent and senescent repeats were highly correlated, with *r*^2^-values of 0.986 and 0.998, respectively. In total, these results suggest that these high-throughput sequencing libraries were highly reproducible and were differentiated from one another based on the biological differences of pre- and post-senescent astrocytes.

We then performed a differential expression analysis that revealed significant senescence-associated changes in the tran-scriptome. Overall, there were 3569 significantly differentially expressed transcripts (*p*_adj_ < 0.05), which represents 18.3% of the total number of detected transcripts, with 1772 transcripts being downregulated in senescence and 1797 transcripts upregulated in senescence (**Figure 1B**). These results demonstrate that there are significant changes to the astrocyte transcriptome during oxidative stress-induced senescence.

### Gene Ontology Term Enrichment Analysis and Tissue Expression of DE Genes

**FIGURE 2:**
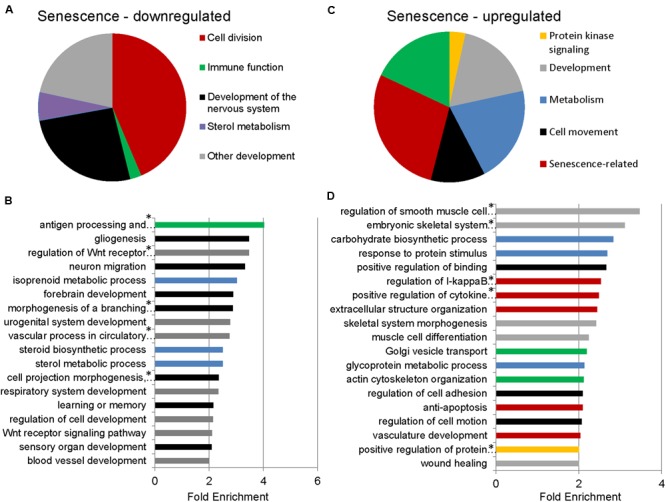
**Gene Ontology (GO) term enrichment analysis for senescent astrocyte down- and upregulated transcripts. (A)** The top overrepresented GO biological process classes in astrocyte downregulated transcripts (*p*_adj_ < 0.05 and fold change of 1.5 or greater; Supplementary Table S3) are shown in the pie chart. The weighted number of genes in each category corresponds to the width of the wedges in the pie chart. **(B)** GO terms that are not cell division related are expanded below in a bar graph. Developmental categories are coded in gray, immune function is in green, sterol metabolism in blue, and development of the nervous system is in black. **(C)** The top overrepresented GO biological process classes by fold enrichment within astrocyte upregulated transcripts (*p*_adj_ < 0.05 and fold change of 1.5 or greater; Supplementary Table S4). **(D)** All GO terms overrepresented by the senescence upregulated genes, ranked by fold enrichment, color coded as in **(C)**. *Full names of GO terms: antigen processing and presentation of peptide or polysaccharide antigen via major histocompatibility complex (MHC) class II, regulation of Wnt receptor signaling pathway, morphogenesis of a branching structure, the combined term of “cell projection morphogenesis, cell morphogenesis involved in differentiation, neuron differentiation,” regulation of smooth muscle cell proliferation, embryonic skeletal system development, regulation of I-kappaB kinase/NF-kappaB cascade, positive regulation of cytokine production, positive regulation of protein kinase cascade.

To identify functional categories of differentially expressed transcripts in senescent astrocytes, we performed GO enrichment analysis using biological process terms with the functional annotation-clustering tool in the DAVID, using a cut-off of 1.5-fold differential expression to define up- or downregulated genes. 1510 downregulated and 1258 upregulated transcripts satisfied this criterion with *p*_adj_ < 0.05, (Supplementary Table S2). Genes involved in cell division, major histocompatibility complex (MHC) class II antigen processing and presentation, metabolism, and CNS development and differentiation were enriched among the downregulated transcripts in senescent astrocytes (**Figures 2A,B**; Supplementary Table S3). MHC Class II presentation and gliogenesis were the two most enriched non-cell division related processes formed by the senescence downregulated transcripts, with enrichment scores of 4 and 3.5, respectively. Among the upregulated gene GO categories, several have known associations with senescence (**Figures 2C,D**; Supplementary Table S4). Inflammation, modification of the extracellular matrix and resistance to apoptosis are known senescence-associated changes (Yoon et al., 2004; Hampel et al., 2006; Freund et al., 2010; Childs et al., 2014) and are represented by the GO terms regulation of I-kappaB kinase/NF-kappaB cascade, positive regulation of cytokine production, extracellular structure organization, vasculature development, and resistance to apoptosis. Cellular adhesion (regulation of cell adhesion, regulation of cell motion, positive regulation of binding) and cytoskeleton (actin cytoskeleton organization) related genes were also previously seen to be upregulated in *in vitro* senescence of human dermal fibroblasts (Yoon et al., 2004). The upregulation of inflammatory genes suggests a mechanism by which astrocyte senescence may be causing further damage in the brain.

In order to identify possible regulators of the senescence-associated genes, we analyzed the promoter regions of differentially regulated genes for over-represented transcription factor binding motifs. GO categories formed by downregulated genes yielded a total of 13 motifs, while those formed by upregulated genes yielded only 1 motif, that for p53, formed by the genes in the ‘extracellular structure organization’ GO category (Supplementary Table S5).

**FIGURE 3:**
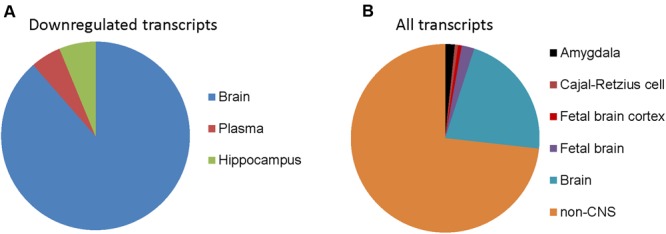
**Tissue expression of detected transcripts in senescent astrocytes. (A)** Tissue annotation expression for astrocyte 1.5-fold or more downregulated transcripts using DAVID (controlling for an FDR at 10%). The width of wedges corresponds to the number of astrocyte downregulated transcripts with tissue expression annotation information that are expressed in the indicated tissues. **(B)** Tissue expression annotation for all transcripts detected in astrocytes.

To determine whether genes with differential expression in oxidative stress-induced astrocyte senescence are brain-expressed, we analyzed differentially expressed transcripts (*p*_adj_ < 0.05) that also had a ≥1.5-fold change, for tissue expression using the UniProt tissue expression database (DAVID:UP_TISSUE). Of all the non-exclusive expression sites found (FDR <10%) for downregulated transcripts, genes belonging to CNS sites comprise the vast majority (762 transcripts or 94.8%), with expressed tissue definition of brain and hippocampus (**Figure 3A**). Therefore, upon the induction of senescence in astrocytes, we see a loss of brain-expressed genes. In contrast, none of the genes upregulated in senescence was CNS-enriched (not shown). CNS enrichment for all detected transcripts was 27%, which is the ratio of the total size of the CNS expression gene sets (7858) to all the defined tissue expression gene sets (29348, FDR <10%, **Figure 3B**). Thus, H_2_O_2_ induced astrocyte senescence specifically downregulates genes that are CNS-enriched.

### Validation of RNA-Seq by qRT-PCR

#### Astrocyte-Enriched Genes

**FIGURE 4:**
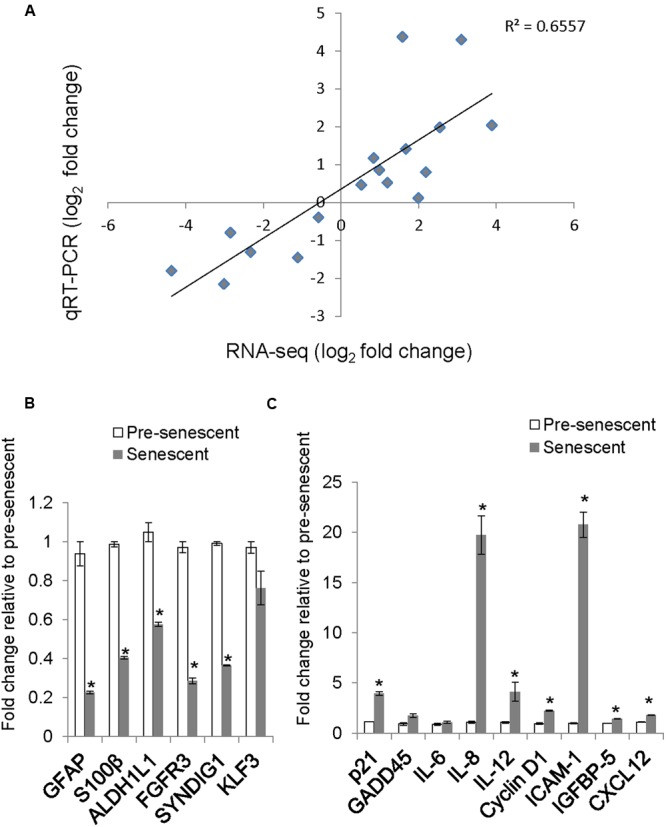
**Validation of selected genes by quantitative real-time PCR (qRT-PCR) and comparison with RNA-Seq log_2_ fold change. (A)** A scatter plot of pre-senescence to senescence log_2_-fold changes obtained by RNA-Seq and qRT-PCR for selected transcripts. Each symbol represents a single transcript, *n* = 17, Pearson *r* = 0.809, (*p* < 0.001 95% CI 0.659 to 0.939). **(B)** Relative levels of astrocyte/brain enriched-genes. Error bars indicate SEM of two independent samples. **(C)** Relative levels of senescence or SASP-related transcripts. Error bars indicate SEM of two independent samples. ^∗^indicates *p* < 0.05, student’s *t*-test.

To determine whether our astrocyte transcriptome data is similar to previously published astrocyte gene expression data, we compared our list of differentially expressed transcripts with cell-type specific markers of astrocytes as described in a previous microarray study (Cahoy et al., 2008). The expression levels of selected astrocyte-enriched genes GFAP, S100B, ALDH1L1, FGFR3, CNS enriched Synapse Differentiation Induced Gene 1 (SynDIG1) and a non-CNS enriched gene, KLF3 were validated by qRT-PCR. The expression of astrocyte and CNS enriched genes was lost or diminished in senescent astrocytes, while that of KLF3 did not change (**Figure 4B**). We verified the association of this decrease in GFAP with senescence by measuring the levels of this protein in pre-senescent astrocytes [cumulative population doubling (cPD) 8.1] and astrocytes that reached replicative senescence, as verified by cessation of growth (cPD 12.2). The GFAP expression in pre-senescent astrocytes was greatly reduced in replicative senescence, confirming our findings with oxidative stress-induced senescence (Supplementary Figure S1). When comparing the log_2_-fold changes in transcript levels between pre-senescent and senescent astrocytes using qRT-PCR and RNA-Seq, we observed a significant positive correlation (*r^2^* = 0.656, *n* = 17) between the results from the two distinct methodologies (**Figure 4A**; Supplementary Table S6). Thus, the loss of astrocyte-enriched genes, combined with the GO analysis demonstrating reduced expression of genes involved in glial and neuronal development suggests loss of normal function in these cells upon undergoing oxidative stress-induced senescence.

#### Senescence-Enriched Genes

We validated by qRT-PCR the levels of senescence-related transcripts that were differentially expressed by RNA-Seq. The levels of senescence-related transcripts *CCND1* (Cyclin D1), *IL8, IGFBP5*, and *ICAM-1* were significantly increased (**Figure 4C**) and correlated with changes observed with RNA-Seq (**Figure 4A**; Supplementary Table S6).

Treatment with H_2_O_2_ to induce senescence robustly induces p21 expression in human diploid fibroblasts (Chen et al., 1998). Surprisingly, the expression of *CDKN1A*, which encodes for the cyclin-dependent kinase inhibitor p21, was not called as significantly differentially expressed in our dataset, although a trend toward increased expression in senescent astrocytes was apparent (RNA-Seq, fold change = 5.84, *p*_adj_ = 0.11). One potential reason for this is low levels of read coverage for this transcript; therefore, we determined the mRNA expression level of p21 using qRT-PCR (**Figure 4C**). We confirmed an almost fourfold increase in p21 mRNA in senescent astrocytes compared with pre-senescent controls.

### Cell Cycle Analysis

**FIGURE 5:**
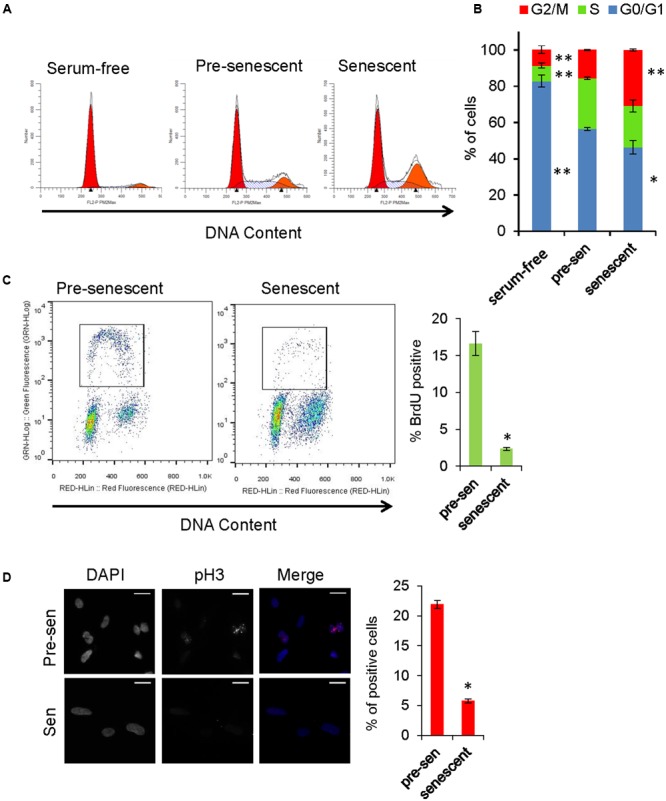
**Cell cycle analysis of senescent astrocytes. (A)** Representative cell cycle profiles of subconfluent pre-senescent astrocytes that were serum-starved, kept in complete growth media, or kept in complete growth media and treated with H_2_O_2_ (200 μM) to induce senescence 7 days prior to staining for DNA content and flow cytometric analysis. Arrowheads indicate, left: Diploid (2N), right: tetraploid (4N) DNA content. **(B)** Graph for percentage of cells in G0/G1 (blue), S (green), or G2/M (red) phases of the cell cycle, that is representative of at least four independent experiments. ^∗^*p* < 0.02, ^∗∗^*p* < 0.001 vs. pre-senescent in growth medium using one-way ANOVA followed by Bonferroni *post hoc* testing. **(C)** Pre-senescent and senescent astrocytes were stained for DNA content and BrdU incorporation. The BrdU-positive population was significantly reduced in senescent astrocytes compared with pre-senescent controls ^∗^*p* < 0.01, Student’s *t*-test. **(D)** Representative images of immunofluorescence staining for mitosis marker phosphorylated histone H3 (pH3) (red) and DAPI (blue), with percent of cells staining positive for pH3 shown in the bar graph. ^∗^*p* < 0.01, student’s *t*-test.

Gene Ontology analysis revealed that genes involved in cell cycle, cell division, and mitosis were over-represented among the downregulated genes in senescent astrocytes consistent with the lost proliferative potential of these cells (“cell division” category, **Figure 2A**). In order to examine the cell cycle distribution of senescent astrocytes, cells were stained for DNA content 7 days after H_2_O_2_ treatment and flow cytometric analysis was performed. Pre-senescent astrocytes that were serum-starved for 24 h arrested predominantly in G0/G1, while in senescent astrocyte cultures, we observed an increase in the fraction of cells with 4N DNA content and a concomitant loss of cells in G0/G1 compared with pre-senescent controls cultured in complete growth medium (**Figures 5A,B**).

The proliferative arrest associated with the onset of cellular senescence has often been presumed to occur solely in G1; however, replicatively senescent cells retain the capacity to synthesize DNA under certain conditions and accumulate in both G1 and G2/M (Mao et al., 2012). A multi-phase cell cycle arrest is also a feature of many cell types exposed to oxidative stress and DNA damage (Baus et al., 2003; Oyama et al., 2011). In order to address the possibility that senescent cells with G2 DNA content are progressing to mitosis, we stained astrocytes for phosphorylated histone H3 (Ser10), which is a marker of mitotic chromosome condensation (Hendzel et al., 1997). Compared with pre-senescent controls, H_2_O_2_-treated astrocytes exhibited few phospho-H3-positive cells (**Figure 5D**). In pre-senescent and senescent astrocyte cultures, we observed a similar proportion of cells with DNA content between 2N and 4N; therefore, we pulsed the cells with BrdU to determine whether they were actively synthesizing DNA. The BrdU-positive population was significantly reduced in senescent astrocytes compared with pre-senescent controls (**Figure 5C**). Overall, these results support a multi-phase cell cycle arrest in H_2_O_2_-induced senescence in human astrocytes.

## Discussion

In order to better understand how astrocyte senescence is linked to aging-related decline in cognition and neurodegeneration, an unbiased interrogation of the changes that occur at the molecular level is essential. Here, we report a comprehensive analysis of the astrocyte transcriptome following the induction of senescence by oxidative stress. Although gene expression changes have been profiled extensively in brain tissue homogenates from different brain regions during aging (Wood et al., 2013) and in Alzheimer’s disease (Twine et al., 2011), fewer studies have addressed cell-type specific changes in these contexts (Simpson et al., 2011; Orre et al., 2014; Sekar et al., 2015). To our knowledge, this is the first report of senescence-associated gene expression changes in a CNS-derived cell type using a whole transcriptome sequencing method (RNA-Seq), which is an accurate and quantitative measurement of transcript abundance.

As expected from the cessation of cell cycle in senescence, the majority of genes downregulated in astrocyte senescence following oxidative stress were related to the cell cycle. Several upregulated genes were also related to senescence-associated phenotypes, such as chronic inflammation (comprising *NFkB activation* and *cytokine production*), extracellular remodeling, and changes in cell morphology (actin cytoskeleton organization).

We found that oxidative stress-induced astrocyte senescence is accompanied by a loss of brain-expressed transcripts involved in neuronal and glial differentiation and development, axonogenesis, and axon guidance. These results are supported by studies of *in vitro* aging in astrocytes where prolonged culture of astrocytes results in a decline in their functional properties including a loss of neuroprotective capacity (Pertusa et al., 2007); and in impaired synaptic transmission in co-culture with neurons (Kawano et al., 2012). The loss of differentiated function upon senescence is also a feature of human ocular keratocytes (Kipling et al., 2009).

The expression of classical markers of astrocyte reactivity —glial fibrillary acidic protein (GFAP) and S100β— is down-regulated with oxidative stress-induced astrocyte senescence in our study. Interestingly, this finding correlates with recent transcriptome analyses showing a decrease in GFAP expression in astrocytes isolated from the brains of aged mice (Orre et al., 2014) and in aged rat cortical tissue homogenates (Wood et al., 2013). Although aging in astrocytes has traditionally been synonymous with an increase in GFAP expression (Pertusa et al., 2007), recent studies have highlighted the heterogeneity of astrocyte expression of stereotypical markers, including GFAP and S100β, in different brain regions during aging (Rodríguez et al., 2014). Furthermore, the response of astrocytes to different CNS insults, in a process termed reactive astrogliosis, is also more heterogeneous than was once thought (Anderson et al., 2014). Although astrocyte senescence shares some features of reactive astrogliosis including cell hypertrophy and the production of inflammatory mediators, whether astrocyte senescence and reactive astrogliosis are distinct phenomena or part of a continuum of changes will require a more comprehensive analysis of these two phenotypes. It is possible that downregulation of certain markers of astrogliosis helps limit the damaging effects of gliosis, or, alternatively, the downregulation may reflect an inability of senescent astrocytes to respond properly to injury. The upregulation of several cytokines and pro-inflammatory genes, on the other hand, suggests that while astrocyte function is decreased in oxidative stress-induced senescence, the cells may be inducing a more general pro-inflammatory environment. The upregulation of Golgi vesicle transport related genes in senescence (**Figure 2**) suggests an increase in the rate of vesicle secretion, which, together with the above categories, would contribute to the SASP. Ablation of reactive astrocytes with upregulated GFAP and vimentin expression, or deletion of these proteins in knockout models have resulted in increased neurodegeneration and immune cell infiltration in models of spinal cord injury and infantile neuronal ceroid lipofuscinosis, respectively (Faulkner et al., 2004; Macauley et al., 2011), supporting a protective role for reactive astrocytes. The decreased GFAP and S100β expression seen in senescent astrocytes may be a contributing factor to neurodegenerative conditions that arise with age.

Astrocyte senescence may be downregulating certain astrocyte immune functions, and in this sense it would be different from astrogliosis. Senescence induced by oxidative stress in astrocytes downregulates the expression of genes involved in antigen processing and presentation on MHC class II proteins. These results are in concordance with a recent RNA-Seq dataset from rat cerebral cortex, which demonstrates a significant downregulation of MHC class II genes (*Cd74*, RT1-ba, *RT1-Da*, and *RT1-Db1*) during aging (Wood et al., 2013). Human homologs of these genes were also downregulated significantly in oxidative stress-induced astrocyte senescence (Supplementary Figure S2). In contrast, mRNA levels of MHC class II genes are elevated in the rat hippocampus with normal aging, suggesting regional differences (Frank et al., 2006). MHC class I and II genes are upregulated in astrocytes isolated from aged mouse cortex (Orre et al., 2014), however, this trend is reversed for MHC class II in the microglial population, suggesting that overall gene expression changes seen in whole brain regions may not be representative of every cell type. In the human brain, a decrease in both GFAP and MHC class II receptors was also observed by immunostaining in the temporal cortex of aged AD subjects (>80 years) compared with younger AD subjects (<80 years; Hoozemans et al., 2010). Furthermore, SNPs in the MHC class II region have been strongly associated with AD in a recent meta-analysis of GWAS studies (Alperovitch et al., 2013), suggesting potentially important functional links to AD pathology.

Although human astrocytes undergo inducible expression of MHC class II antigens, their role as functional antigen presenting cells is controversial (Jensen et al., 2013); therefore, the functional significance of a loss of MHC class II gene expression in senescent astrocytes is unclear. In professional antigen-presenting cells, activation of p38MAPK has been shown to negatively regulate *CIITA*, the master regulator of MHC class II gene expression (Yao et al., 2006). Because p38 MAPK activation is a key pathway driving senescence, this suggests a possible convergence between the senescence program (Iwasa et al., 2003; Bhat et al., 2012) and dysregulation of immune function during aging or immunosenescence. Consistent with this idea, inducible MHC class II expression is impaired during aging in murine macrophages (Herrero et al., 2001).

There are also parallels between gene expression changes in astrocytes with AD and our senescence RNA-Seq data, as expected from the increase of senescent astrocytes in AD brain (Bhat et al., 2012). We compared senescence gene expression changes *in vitro* to those in astrocytes captured by laser capture microdissection from brains of deceased subjects with early or late stage AD, as analyzed by microarray in a previously published study (Simpson et al., 2011). Thirty-one genes showed a decrease greater than 1.5-fold in both astrocyte senescence *in vitro* and in astrocytes in AD (Supplementary Table S7). Seven GO terms were significantly represented (FDR < 10%) by the genes downregulated in senescence and AD, out of which four were related to development of non-CNS organs. The remaining three GO categories were *neuron development, cell–cell signaling*, and *neuron differentiation* (Supplementary Table S8). The fold changes for the genes in these GO categories are shown in Supplementary Figure S3.

We thus observe that genes involved in generation and differentiation of neural cell types were commonly down-regulated in astrocytes in oxidative stress induced senescence and in Alzheimer’s disease. Among these genes, the neurotrophic tyrosine kinase 2 receptor (NTRK2) gene codes for the tyrosine kinase B receptor (TrkB). TrkB’s primary ligand is brain-derived neurotrophic factor (BDNF) and its phosphorylation activates pathways involved in neuronal survival, growth, differentiation, transmission, and synaptic plasticity (Boulle et al., 2012). Expression of NRTK2 was also lower in neurons from the anterior cingulate cortex of brains from patients with autism spectrum disorder (Chandley et al., 2015). Another gene with known CNS function that is represented in these GO terms is FGF9. Knockdown of FGF9 downregulates astrogenesis in the developing rat brain and when added to *ex vivo* cultures, FGF9 upregulates this process (Falcone et al., 2015). FGF9 conditional knockdown caused movement and growth defects in mice, with defects in Bergmann glia formation and Purkinje cell alignment possibly due to a lack of signals from the Bergmann glia. Moreover, extracellular FGF9 was shown to be necessary for glia to form radial morphology. (Lin et al., 2009). Other genes related to neural regeneration and development that are not included in these GO terms were also commonly downregulated between oxidative stress-induced astrocyte senescence and AD (Supplementary Table S7). One such gene, teneurin transmembrane protein 4 (TENM4), encodes for the teneurin-4 (Ten-4) transmembrane protein. An insertion into this gene was responsible for tremors in mice and caused defects in myelination of small diameter axons. The cause was shown to be inhibited oligodendrocyte differentiation, growth and process formation, due to defective FAK signaling by Ten-4 (Suzuki et al., 2012). Ten-4 overexpression and knockdown experiments have shown that this protein is necessary for filopodia formation and neurite outgrowth in neurons via FAK and N-WASP signaling (Suzuki et al., 2014). An intronic variant of this gene was also significantly overrepresented in genomes of bipolar disorder patients (Witt et al., 2014). The protein product (γ-1-syntrophin) of another gene downregulated in senescence and AD, SNTG1, binds and localizes the neurotrophic peptide γ–enolase to the plasma membrane and neurite growth cones of neuroblastoma cells. Knockdown of γ-1-syntrophin disrupts this localization and inhibits the neurite outgrowth and cell proliferation induced by exogenous γ–enolase peptide (Hafner et al., 2010; Falcone et al., 2015). Furthermore, numerous observations of senescence markers in mammalian development may explain the abundance of GO terms related to development of other tissues in the senescence up- and downregulated genes (Meisler and Paigen, 1972; Barral et al., 2014). These gene classes are also an important part of the total down regulated transcriptome in oxidative stress-induced astrocyte senescence. These findings suggest that senescence may be contributing to AD through slowing down of regeneration and differentiation of astrocytes and neurons. Changes in neurogenesis rates were indeed observed in multiple animal and *in vitro* models of AD (Winner and Winkler, 2015).

We define the transcriptional response of human astrocytes to H_2_O_2_ induced senescence, which has unique characteristics compared to that of other cell types. Whereas H_2_O_2_ induced senescence led to three times as many upregulated genes as downregulated genes in a human hepatocyte cell line (Aravinthan et al., 2014), the number of downregulated genes was slightly higher for astrocyte senescence (Supplementary Figure S4). There were significantly more genes regulated in the same direction by senescence in both cell types than would be expected by chance, however, the differentially regulated gene sets from the two cell types are clearly distinct. These findings suggest cell-type specific responses to oxidative stress induced senescence, with shared mechanisms.

Aging is a major risk factor for chronic diseases in a host of organ systems. The clearance of senescent cells alleviates several signs of pathology associated with aging (Baker et al., 2011, 2016), suggesting that the presence of senescent cells may be deleterious for tissue and organism homeostasis. There is now strong evidence that senescent cells accumulate in tissues, including brain, during aging and in the setting of pathology. We propose that oxidative stress-induced astrocyte senescence is a model for understanding how the basic processes of aging may lead to a decline in cognition and neurodegeneration, and for identification of potential targets for therapeutic intervention.

## Author Contributions

Conceived and designed experiments EC, FT, BG, GD, SG, CS, FJ, and CT. Perform the experiments EC, FT, BG, SG, and CS. Analyzed the data EC, FT, BG, GD, SG, YL, EY, JC, RN, L-SW, NB, SB, FJ, and CT. Contributed reagents/materials/analysis tools BG, GD, SG, YL, EY, JC, RN, L-SW, CS, NB, SB, FJ, and CT. Wrote the manuscript EC, FT, BG, GD, YL, FJ, and CT.

## Conflict of Interest Statement

The authors declare that the research was conducted in the absence of any commercial or financial relationships that could be construed as a potential conflict of interest.

## Footnotes

1 genome.ucsc.edu/ENCODE/protocols/dataStandards/ENCODE_RNAseq_Standards_V1.0.pdf

2 https://genome.ucsc.edu/

3 http://cistrome.org/ap/root
