# Supplementary material for: Changes in the Transcriptome of Human Astrocytes Accompanying Oxidative Stress-Induced Senescence
Source: Front Aging Neurosci. 2016 Aug 31;8:208. doi: 10.3389/fnagi.2016.00208 (PMC5005348; doi:10.3389/fnagi.2016.00208)
Supplement: Supplementary file 9 [file Presentation_1.PPTX]

## Slide 1
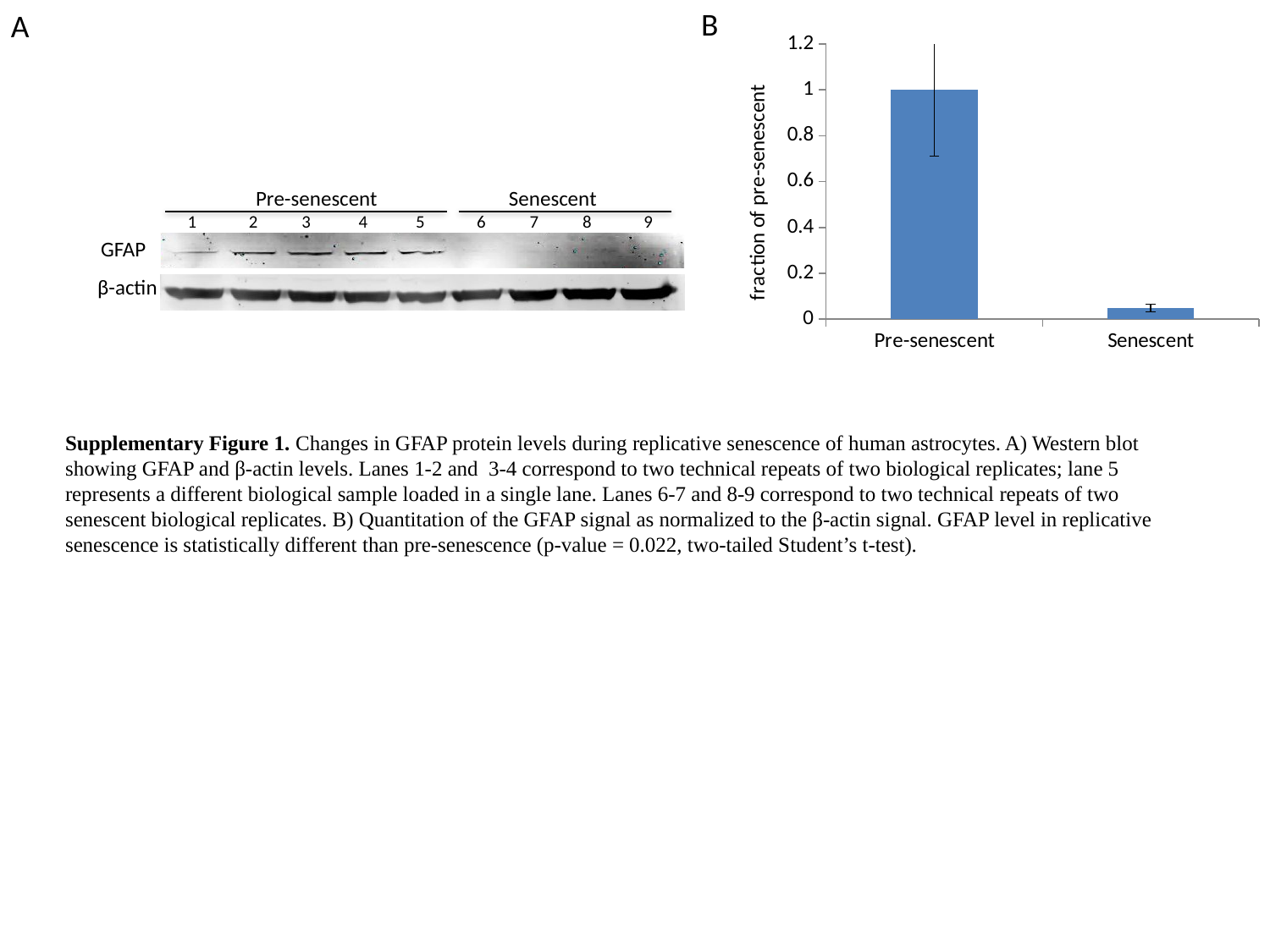

A
B
### Chart
| Category | |
|---|---|
| Pre-senescent | 1.0 |
| Senescent | 0.0488332283103369 |Pre-senescent 	 Senescent
GFAP
β-actin
1 2 3 4 5 6 7 8 9
Supplementary Figure 1. Changes in GFAP protein levels during replicative senescence of human astrocytes. A) Western blot showing GFAP and β-actin levels. Lanes 1-2 and 3-4 correspond to two technical repeats of two biological replicates; lane 5 represents a different biological sample loaded in a single lane. Lanes 6-7 and 8-9 correspond to two technical repeats of two senescent biological replicates. B) Quantitation of the GFAP signal as normalized to the β-actin signal. GFAP level in replicative senescence is statistically different than pre-senescence (p-value = 0.022, two-tailed Student’s t-test).
