# Supplementary material for: Changes in the Transcriptome of Human Astrocytes Accompanying Oxidative Stress-Induced Senescence
Source: Front Aging Neurosci. 2016 Aug 31;8:208. doi: 10.3389/fnagi.2016.00208 (PMC5005348; doi:10.3389/fnagi.2016.00208)
Supplement: Supplementary file 10 [file Presentation_2.PPTX]

## Slide 1
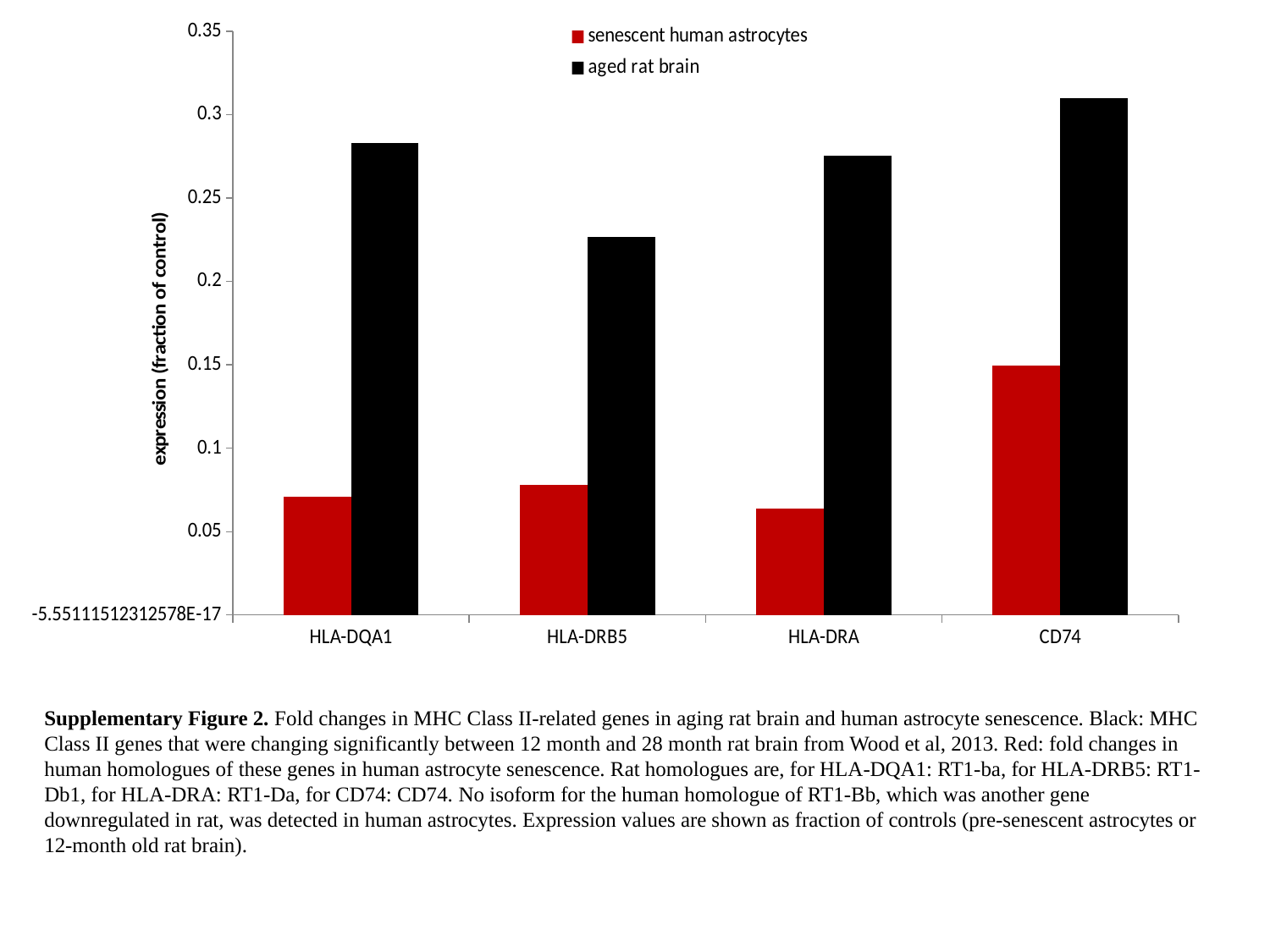

### Chart
| Category | senescent human astrocytes | aged rat brain |
|---|---|---|
| HLA-DQA1 | 0.0709867482146351 | 0.28322097132395 |
| HLA-DRB5 | 0.0779436048583513 | 0.22687978882929 |
| HLA-DRA | 0.0637290299703748 | 0.275476278969153 |
| CD74 | 0.149764670334757 | 0.309926924984747 |Supplementary Figure 2. Fold changes in MHC Class II-related genes in aging rat brain and human astrocyte senescence. Black: MHC Class II genes that were changing significantly between 12 month and 28 month rat brain from Wood et al, 2013. Red: fold changes in human homologues of these genes in human astrocyte senescence. Rat homologues are, for HLA-DQA1: RT1-ba, for HLA-DRB5: RT1-Db1, for HLA-DRA: RT1-Da, for CD74: CD74. No isoform for the human homologue of RT1-Bb, which was another gene downregulated in rat, was detected in human astrocytes. Expression values are shown as fraction of controls (pre-senescent astrocytes or 12-month old rat brain).
