# Supplementary material for: Changes in the Transcriptome of Human Astrocytes Accompanying Oxidative Stress-Induced Senescence
Source: Front Aging Neurosci. 2016 Aug 31;8:208. doi: 10.3389/fnagi.2016.00208 (PMC5005348; doi:10.3389/fnagi.2016.00208)
Supplement: Supplementary file 11 [file Presentation_3.PPTX]

## Slide 1
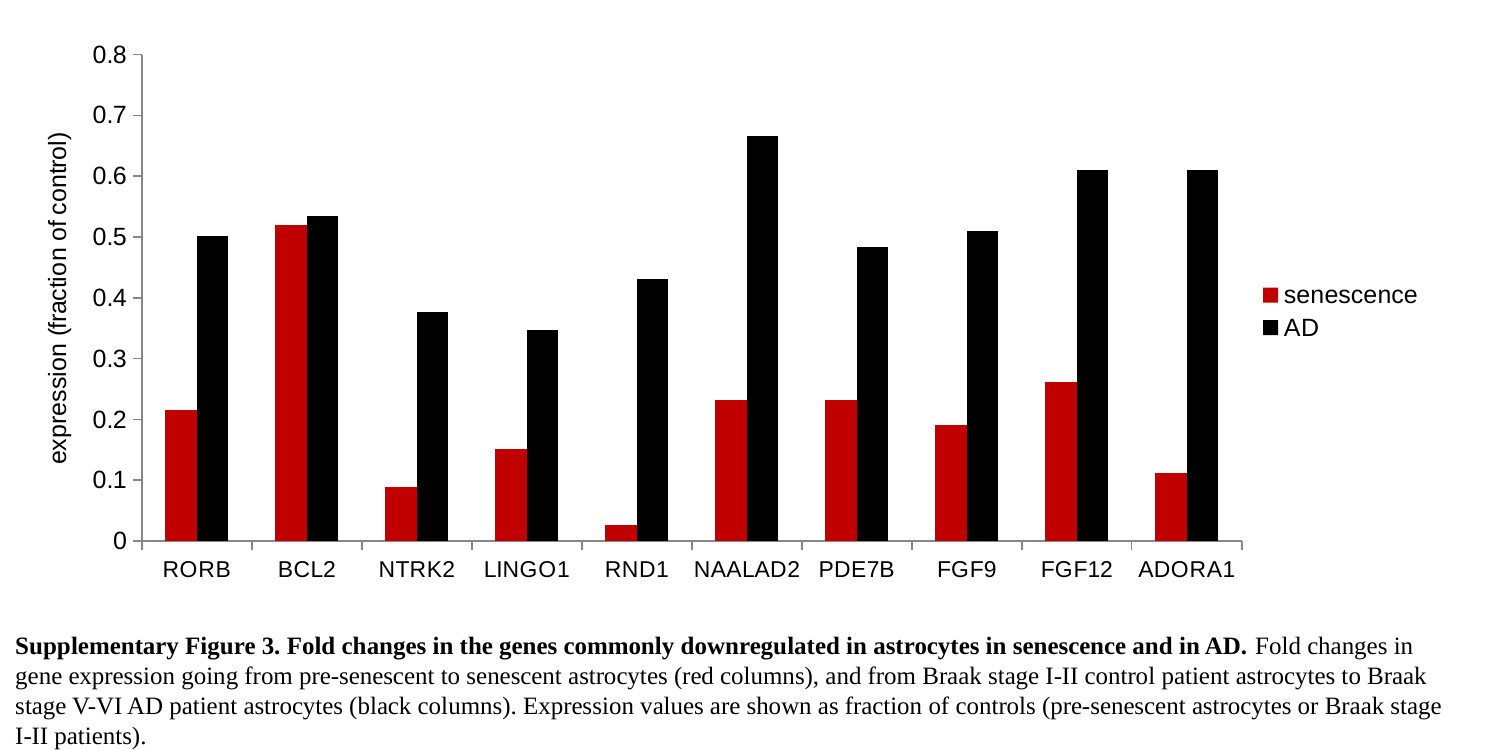

### Chart
| Category | senescence | AD |
|---|---|---|
| RORB | 0.215202437746551 | 0.50251256281407 |
| BCL2 | 0.520561949102933 | 0.53475935828877 |
| NTRK2 | 0.0882714538825376 | 0.37593984962406 |
| LINGO1 | 0.150787729242248 | 0.347222222222222 |
| RND1 | 0.0261755225071852 | 0.431034482758621 |
| NAALAD2 | 0.231251532666711 | 0.666666666666667 |
| PDE7B | 0.231124670331366 | 0.483091787439614 |
| FGF9 | 0.190007439645125 | 0.510204081632653 |
| FGF12 | 0.261652230525224 | 0.609756097560976 |
| ADORA1 | 0.112315701748022 | 0.609756097560976 |Supplementary Figure 3. Fold changes in the genes commonly downregulated in astrocytes in senescence and in AD. Fold changes in gene expression going from pre-senescent to senescent astrocytes (red columns), and from Braak stage I-II control patient astrocytes to Braak stage V-VI AD patient astrocytes (black columns). Expression values are shown as fraction of controls (pre-senescent astrocytes or Braak stage I-II patients).
