# Supplementary material for: Changes in the Transcriptome of Human Astrocytes Accompanying Oxidative Stress-Induced Senescence
Source: Front Aging Neurosci. 2016 Aug 31;8:208. doi: 10.3389/fnagi.2016.00208 (PMC5005348; doi:10.3389/fnagi.2016.00208)
Supplement: Supplementary file 12 [file Presentation_4.PPTX]

## Slide 1
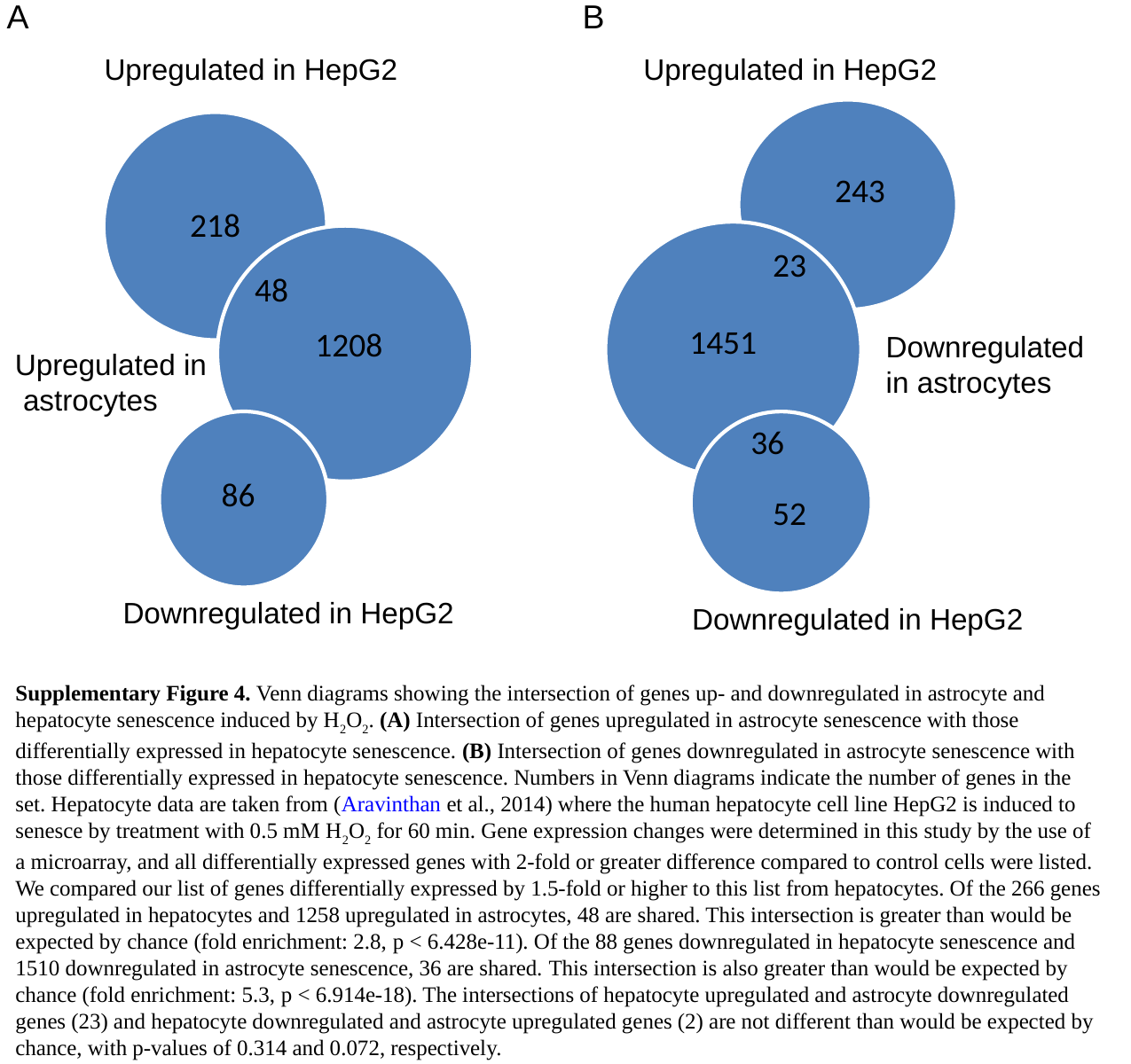

A
B
Upregulated in HepG2
Upregulated in HepG2
243
218
23
48
1451
1208
Downregulated
in astrocytes
Upregulated in
 astrocytes
2
36
86
52
Downregulated in HepG2
Downregulated in HepG2
Supplementary Figure 4. Venn diagrams showing the intersection of genes up- and downregulated in astrocyte and hepatocyte senescence induced by H2O2. (A) Intersection of genes upregulated in astrocyte senescence with those differentially expressed in hepatocyte senescence. (B) Intersection of genes downregulated in astrocyte senescence with those differentially expressed in hepatocyte senescence. Numbers in Venn diagrams indicate the number of genes in the set. Hepatocyte data are taken from (Aravinthan et al., 2014) where the human hepatocyte cell line HepG2 is induced to senesce by treatment with 0.5 mM H2O2 for 60 min. Gene expression changes were determined in this study by the use of a microarray, and all differentially expressed genes with 2-fold or greater difference compared to control cells were listed. We compared our list of genes differentially expressed by 1.5-fold or higher to this list from hepatocytes. Of the 266 genes upregulated in hepatocytes and 1258 upregulated in astrocytes, 48 are shared. This intersection is greater than would be expected by chance (fold enrichment: 2.8, p < 6.428e-11). Of the 88 genes downregulated in hepatocyte senescence and 1510 downregulated in astrocyte senescence, 36 are shared. This intersection is also greater than would be expected by chance (fold enrichment: 5.3, p < 6.914e-18). The intersections of hepatocyte upregulated and astrocyte downregulated genes (23) and hepatocyte downregulated and astrocyte upregulated genes (2) are not different than would be expected by chance, with p-values of 0.314 and 0.072, respectively.
